# Downregulation of microRNA-448 inhibits IL-1β-induced cartilage degradation in human chondrocytes via upregulation of matrilin-3

**DOI:** 10.1186/s11658-018-0072-6

**Published:** 2018-02-22

**Authors:** Hao Yang, Di Wu, Hua Li, Nan Chen, Yongjun Shang

**Affiliations:** 1 Department of Orthopedics, First Affiliated Hospital of Kunming Medical University, Kunming, 650032 Yunnan Province People’s Republic of China; 2 Department of Orthopedics, Dalian University Affiliated Xinhua Hospital, No. 156 Xinhua Street, Shahekou District, Dalian, 116021 Liaoning Province People’s Republic of China

**Keywords:** Osteoarthritis, MicroRNA-448, Interleukin-1 beta, Chondrocyte, Extracellular matrix, Matrilin-3

## Abstract

**Background:**

Osteoarthritis is characterized by the continuous degradation of the articular cartilage. The microRNA miR-448 has been found to be broadly involved in cellular processes, including proliferation, apoptosis, invasion and EMT. While aberrant expression of miR-448 has been found in multiple cancers, its level in osteoarthritis cartilage and its role in the progression of this disease are still unknown. Here, we examined the functional roles of miR-448 and its expression in osteoarthritis tissues, including IL-1β-stimulated osteoarthritis chondrocytes.

**Methods:**

Chondrocytes were isolated from human articular cartilage and stimulated with IL-1β. The expression levels of miR-448 in the cartilage and chondrocytes were both determined. After transfection with an miR-448 mimic or inhibitor, the mRNA levels of aggrecan, type II collagen and MMP-13 were determined. Luciferase reporter assay, qRT-PCR and western blot were performed to explore whether matrilin-3 was a target of miR-448. Furthermore, we co-transfected chondrocytes with miR-448 inhibitor and siRNA for matrilin-3 and then stimulated them with IL-1β to determine whether miR-448-mediated IL-1β-induced cartilage matrix degradation resulted from directly targeting matrilin-3.

**Results:**

The level of miR-448 was significantly higher and matrilin-3 expression was significantly lower in osteoarthritis cartilage and IL-1β-induced chondrocytes than in normal tissues and cells. Furthermore, matrilin-3 expression was reduced by miR-448 overexpression. MiR-448 downregulation significantly alleviated the IL-1β-induced downregulation of aggrecan and type II collagen expression, and upregulation of MMP-13 expression. MiR-448 overexpression had the opposite effects. Knockdown of matrilin-3 reversed the effects of the miR-448 inhibitor on the expressions of aggrecan, type II collagen and MMP-13.

**Conclusion:**

The findings showed that miR-448 contributed to the progression of osteoarthritis by directly targeting matrilin-3. This indicates that it has potential as a therapeutic target for the treatment of osteoarthritis.

## Background

Osteoarthritis (OA), which is characterized by articular cartilage degradation, is the most common type of degenerative joint disease [1]. Chondrocytes are the only cells found in the articular cartilage and they play a critical role in maintaining matrix integrity and tissue homeostasis [2]. During the progress of osteoarthritis, they are metabolically activated, whereupon they subsequently disrupt the metabolic homeostasis of the extracellular matrix (ECM) by increasing the production of matrix-degrading enzymes such as matrix metalloproteinase 13 (MMP-13) and decreasing the synthesis of aggrecan and type II collagen [3]. This results in the degradation of the ECM. Developing effective therapeutic strategies to treat osteoarthritis will certainly require an understanding of chondrocyte injuries and related mechanisms.

It has been reported that interleukin-1 beta (IL-1β), one of the main cytokines involved in the pathogenesis of osteoarthritis, can inhibit cell proliferation, reduce the synthesis of aggrecan and type II collagen, and increase the expression of MMP-13 in chondrocytes [2, 3].

MicroRNAs (miRNAs) are a class of non-coding small RNA identified as critical post-transcriptional regulators. They play critical roles in several biological processes, including cell proliferation, migration, invasion and differentiation [4, 5]. An increasing number of studies have indicated that miRNAs participate in the pathogenesis of osteoarthritis, with confirmation for miR-139, miR-140, miR-23a-3p and miR-365 [6–9].

It has been reported that miR-448 is broadly involved in cellular processes, including proliferation, apoptosis, invasion and epithelial–mesenchymal transition (EMT) [10, 11]. Aberrant miR-448 expression has been confirmed in many cancers [12–14]. However, the levels of miR-448 in osteoarthritis cartilage and its role in progression of this disease are still unclear.

Earlier studies have also demonstrated that the expression of matrilin-3 is dramatically lower in osteoarthritis cartilage than in normal cartilage [15]. Thus, miRNAs and matrilin-3 clearly both have the important roles in the pathogenesis of osteoarthritis. The online database microRNA.org was used to predict that matrilin-3 is a binding target of miR-448. This study focuses on whether miR-448 plays a significant role in progression of osteoarthritis by targeting matrilin-3.

Our data show that the miR-448 level significantly increased and the expression of matrilin-3 significantly decreased in osteoarthritis cartilage compared to normal cartilage. Furthermore, we confirmed that miR-448 could directly target the matrilin-3 gene. The functional analysis showed that knockdown of miR-448 upregulated IL-1β-inhibited the expressions of aggrecan and type II collagen, and downregulated IL-1β-induced MMP-13 expression by increasing matrilin-3 expression, contributing to the protection of chondrocytes from IL-1β-induced degradation of ECM. To confirm these results, the functional roles of miR-448 were studied in osteoarthritis chondrocytes. As expected, miR-448 also had similar effects on IL-1β-induced chondrocytes. According to the findings, miR-448 is a promising target for prevention and treatment of osteoarthritis.

## Methods

### Specimen selection

Human cartilage samples were collected from 10 osteoarthritis patients undergoing total knee replacement surgery and from 10 traumatic amputees without rheumatoid arthritis or osteoarthritis. Patients with osteoarthritis were diagnosed according to the American College of Rheumatology criteria. All samples were collected from the Department of Orthopedics, Dalian University Affiliated Xinhua Hospital. Informed consent was obtained from all patients and the study was approved by the Ethics Committee of Dalian University Affiliated Xinhua Hospital.

### Cell culture and treatment

To isolate the primary osteoarthritis and normal chondrocytes, osteoarthritis and normal articular cartilage slices were chopped finely with a scalpel blade. Cartilage tissue was predigested with trypsin (0.1%, *w*/*v*; Sigma) for 10 min at 37 °C. After removing the trypsin solution, the tissue slices were treated overnight with type IV *Clostridium* collagenase in Dulbecco’s modified Eagle medium (DMEM) with 5% fetal bovine serum (FBS). Then the sample was filtered to remove the undigested cartilage and the chondrocyte cells were pelleted at 2000 x g for 5 min before being resuspended. After the initial isolation, the cells were maintained in high-density cultures in DMEM supplemented with 10% FBS, L-glutamine, 100 units/ml penicillin, and 100 μg/ml streptomycin (GIBCO). First passage chondrocytes were obtained after 2 weeks. All the experiments were done within 3 days of the passage 1 culture.

Primary osteoarthritis and normal chondrocytes were transfected with miR-448 mimic or inhibitor (RiboBio) at a 100 nM concentration by using Lipofectamine 3000 reagent (Invitrogen) following the manufacturer’s instructions. After transfection for 48 h, the medium was removed, and cells were stimulated with 5 ng/ml IL-1β for 24 h or were not stimulated, and then total RNA prepared from chondrocytes was used to check the expressions of miR-448, matrilin-3, aggrecan, type II collagen and MMP-13. Cell supernatants were harvested and MMP-13 production was quantified via ELISA.

### RNA extraction and quantitative real-time PCR

Total RNA of chondrocytes was extracted for the analysis of miRNA and mRNA following the manufacturer’s instructions. For quantification of miR-448, the TaqMan MicroRNA Reverse Transcription Kit and TaqMan miRNA assay (Qiagen) were respectively used to perform reverse transcription and PCR following the manufacturer’s instructions. U6 was used as the internal control. The gene expressions of matrilin-3, aggrecan, type II collagen and MMP-13 were detected using the SYBR Green PCR kits (TAKARA). GAPDH served as an internal control. All PCR assays were performed in CFX96 Touch (Bio-Rad). The following primers were used:

- Matrilin-3 forward, 5’-TCTCCCGGATAATCGACACTC-3′, reverse, 5’-CAAGGGTGTGATTCGACCCA-3’
- Aggrecan forward, 5’-ACCAGACGGGCCTCCCAGAC-3′, reverse, 5’-TGGCTCTGCCCCAGAGGGAC-3′
- Type II collagen forward, 5’-TGAGGGCGCGGTAGAGACCC-3′, reverse, 5’-TGCACACAGCTGCCAGCCTC-3’
- MMP-13 forward, 5’-ATGCGGGGTTCCTGATGTGG-3′, reverse, 5’-GGCCCAGGAGGAAAAGCATG-3’
- GAPDH forward, 5’-ACAACTTTGGTATCGTGGAAGG-3′, reverse, 5’-GCCATCACGCCACAGTTTC-3’
- U6 forward, 5’-CGCTTCGGCAGCACATATAC-3′, reverse, 5’-AAATATGGAACGCTTCACGA-3′

### Western blot analysis

Chondrocytes were lysed using RIPA buffer (Beyotime Institute of Biotechnology) with a protease inhibitor cocktail (Millipore). The protein concentration of cell lysates was quantified using a BCA Kit (Beyotime Institute of Biotechnology), and 50 ng of protein were separated via 8% SDS-PAGE and then transferred onto a PVDF membrane (Millipore). The membranes were blocked in 5% non-fat dry milk diluted with TBST at room temperature for 1 h and probed overnight at 4 °C with anti-matrilin-3 antibody (ab106388; 1:1000 dilution; Abcam). After that, the membranes were washed with TBST and incubated with a goat anti-rabbit IgG conjugated to horseradish peroxidase (1:1000 dilution; Abcam) for 1 h at room temperature. Incubation with monoclonal mouse GAPDH antibody (ab9485; 1:5000 dilution; Abcam) was performed as the loading control. The proteins were visualized using ECL western blotting detection reagents (Millipore). The densitometry of the bands was quantified using the Image J 1.38X software.

### Dual-luciferase reporter assay

Before transfection, chondrocytes were seeded in 6-well plates (1 × 10^6^ cells/well) and incubated for 24 h. Next, the wild-type pGL3-matrilin-3-3’UTR or mutant pGL3-matrilin-3-3’UTR plasmid (GeneChem), the miR-448 inhibitor and anti-miR-NC inhibitor, or the miR-448 mimic and miR-NC mimic, and the pRL-TK Renilla luciferase reporter (Promega) were co-transfected into the chondrocytes using Lipofectamine 3000 (Invitrogen). Chondrocytes were collected 48 h after transfection and analyzed using the dual-luciferase reporter assay. Firefly luciferase activities were normalized to renilla luciferase activities.

### MMP-13 ELISA

After transfection, chondrocytes were treated with IL-1β for 24 h, and the expression of MMP-13 in the culture supernatants was quantified using an ELISA kit according to the manufacturer’s instructions (R&D Systems, Inc.). Plates were read at 450 nm using a Thermo Fisher Scientific Microplate Reader, and the MMP-13 concentration in the samples was calculated using a standard curve.

### Statistical analysis

Experiments were repeated at least three times. Values are expressed as means ± SEM. Data were evaluated for statistical significance using one-way analysis of variance (ANOVA) with *p* < 0.05 considered a statistically significant difference. All statistical analyses were performed using GraphPad Prism 5.0 (GraphPad Software, Inc.).

## Results

### The level of miR-448 was increased and the expression of matrilin-3 was decreased in osteoarthritis cartilage and IL-1β-induced chondrocytes

**Fig. 1:**
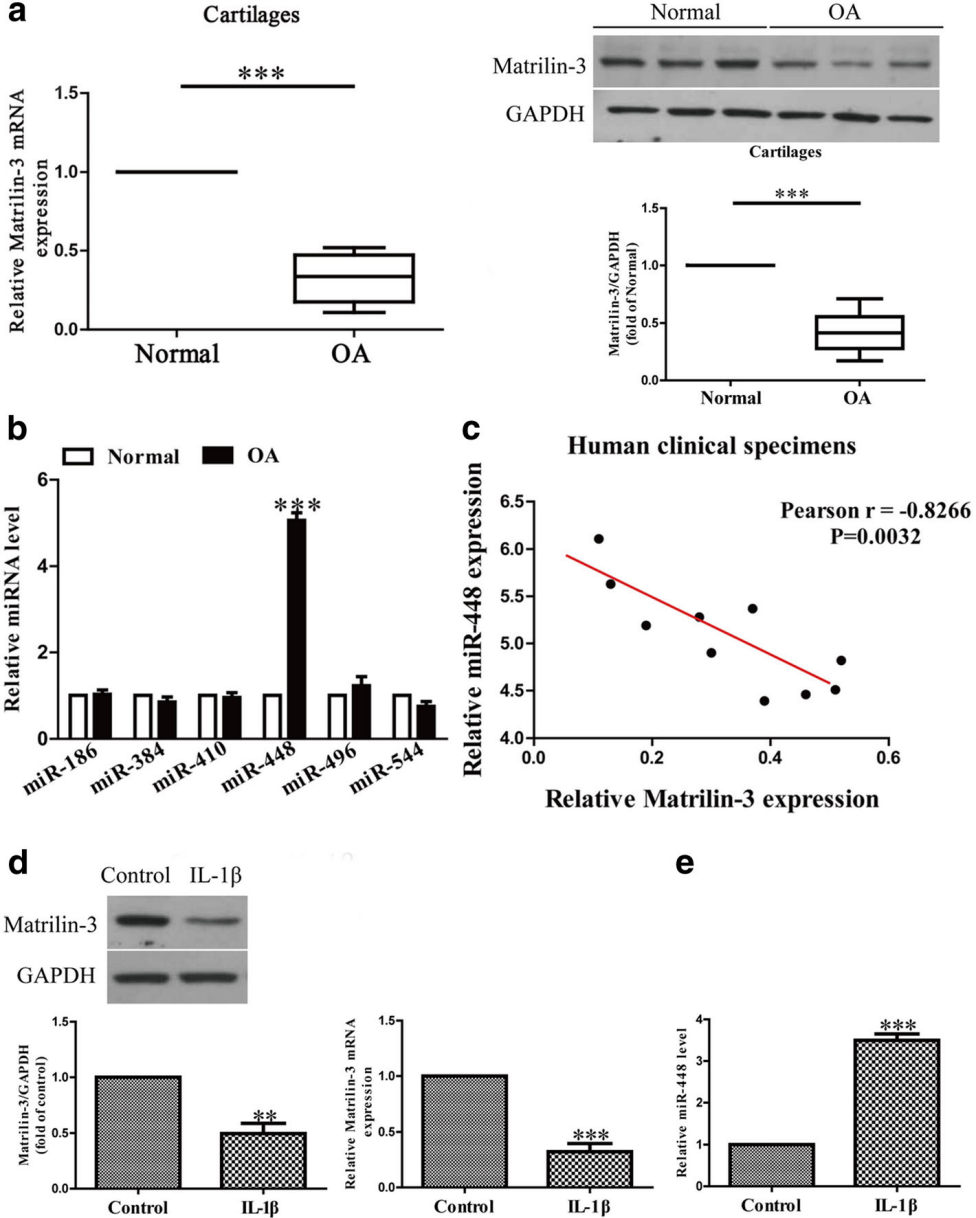
The levels of matrilin-3 and miR-448 in osteoarthritis cartilage and IL-1β-induced chondrocytes. **a** The mRNA and protein levels of matrilin-3 in normal and osteoarthritis cartilages were determined using quantitative real-time PCR and western blot, *n* = 10. **b** The levels of miR-186, miR-384, miR-410, miR-448, miR-496 and miR-544 in osteoarthritis cartilages were determined using quantitative real-time PCR, *n* = 10. **c** Pearson’s correlation analysis of the relative expression levels of miR-448 and the relative matrilin-3 mRNA levels in osteoarthritis cartilage. **d** Chondrocytes were treated with 5 ng/ml IL-1β for 24 h. The expression of matrilin-3 was analyzed using quantitative real-time PCR and western blot, and normalized to GAPDH, *n* = 4. **e** The level of miR-448 was analyzed using quantitative real-time PCR and normalized to U6, *n* = 4. The data shown are means ± SEM. OA: chondrocytes from patients with osteoarthritis. Normal: chondrocytes from patients without osteoarthritis. ***p < 0.001 vs. normal or control

In this study, the mRNA and protein levels of matrilin-3 in osteoarthritis cartilage were respectively detected using quantitative real-time PCR and western blot. Our data demonstrate that the matrilin-3 expression was dramatically reduced in osteoarthritis cartilage (Fig. 1a), which is consistent with previous findings [15].

Based on information obtained using the online database microRNA.org, we choose miR-186, miR-384, miR-410, miR-448, miR-496 and miR-544 to further study which miRNA might regulate matrilin-3 expression. We found that the miR-448 level was dramatically higher in osteoarthritis cartilage than in normal cells (Fig. 1b) and was the highest among these six miRNAs.

To determine whether the matrilin-3 expression was closely related to miR-448 level in osteoarthritis cartilage, Pearson’s correlation analysis was applied. It revealed a significant inverse correlation between matrilin-3 and miR-448 in osteoarthritis cartilage (Fig. 1c).

To investigate the functional roles of matrilin-3 and miR-448 in osteoarthritis, the expressions of matrilin-3 and miR-448 were determined in IL-1β-induced chondrocytes. Compared with the untreated group, the mRNA and protein levels of matrilin-3 in IL-1β-induced chondrocytes were lower (Fig. 1d), and the miR-448 level was higher (Fig. 1e).

### Effects of miR-448 on IL-1β-induced inhibition of aggrecan and type II collagen mRNA expressions in chondrocytes

**Fig. 2:**
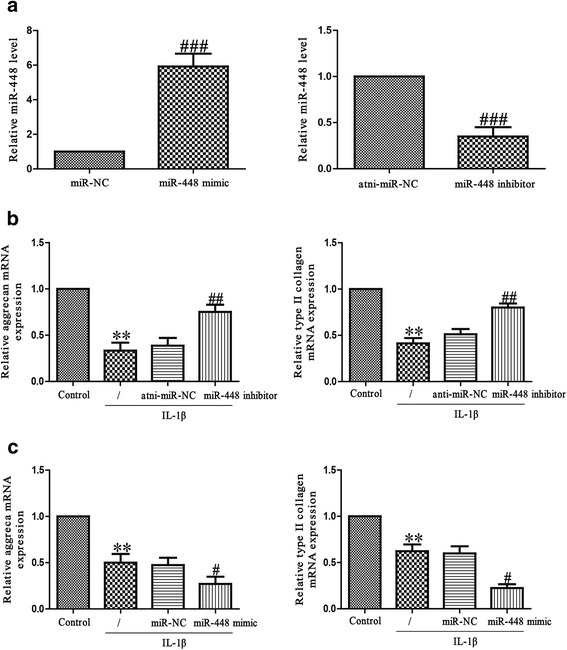
Effects of miR-448 on IL-1β-induced inhibition of aggrecan and type II collagen mRNA expressions inchondrocytes. **a** Chondrocytes were transfected with miR-448 mimic or inhibitor. The level of miR-448 was determined using quantitative real-time PCR. Chondrocytes were treated with 5 ng/ml IL-1β for 24 h after transfection with miR-448 inhibitor **b** or mimic **c**. The mRNA levels of aggrecan and type II collagen were determined using quantitative real-time PCR. The data shown are means ± SEM, *n* = 4. **p < 0.01 vs. control; ^#^*p* < 0.05, ^##^*p* < 0.01, ^###^*p* < 0.001 vs. vehicle + IL-1β

To study the effect of miR-448 on the IL-1β-induced chondrocytes, we transfected chondrocytes with an miR-448 mimic or inhibitor before treatment with IL-1β. The miRNA transfection efficiency was evaluated using quantitative real-time PCR. The miR-448 level was significantly higher in the miR-448 mimic group than in the miR-NC group after transfection for 48 h (Fig. 2a). The level of miR-448 was significantly decreased in chondrocytes transfected with miR-448 inhibitor compared with the cell treated with anti-miR-NC.

Next, to explore whether miR-448 had an effect on IL-1β-induced matrix degradation, we transfected chondrocytes with miR-448 mimic or inhibitor, and then exposed chondrocytes to IL-1β for 24 h. The mRNA levels of aggrecan and type II collagen were analyzed using quantitative real-time PCR. As expected, the aggrecan and type II collagen mRNA levels were dramatically restored in chondrocytes transfected with miR-448 inhibitor when compared with the levels for IL-1β-treated chondrocytes (Fig. 2b). However, miR-448 mimic further reduced aggrecan and type II collagen synthesis in IL-1β-treated chondrocytes (Fig. 2c), further confirming the effect of miR-448 in aggrecan and type II collagen mRNA expressions.

### Effects of miR-448 on IL-1β-induced MMP-13 production in chondrocytes

**Fig. 3:**
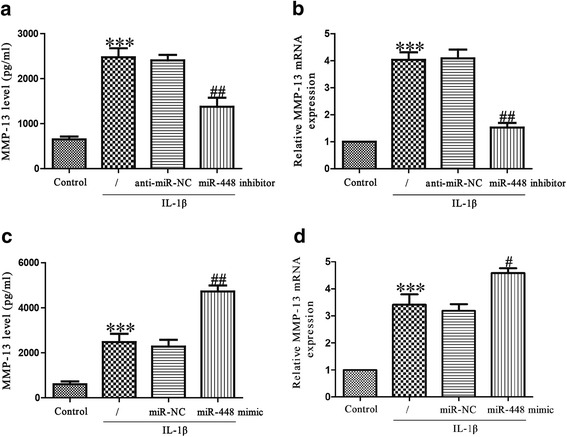
Effect of miR-448 on IL-1β-induced MMP-13 in chondrocytes. Chondrocytes were treated with IL-1β (5 ng/ml) for 24 h after transfection with miR-448 mimic or inhibitor. **a**, **c** The expression level of secreted MMP-13 protein was determined using ELISA. **b**, **d** The mRNA level of MMP-13 was determined using quantitative real-time PCR. The data shown are means ± SEM, *n* = 4. ***p < 0.001 vs. control; ^#^*p* < 0.05, ^##^*p* < 0.01 vs. vehicle + IL-1β

MMP-13 is a major collagenase involved in the catabolic processes in osteoarthritis. It acts by cleaving key ECM constituents such as aggrecan and type II collagen. Chondrocytes were stimulated with IL-1β for 24 h after transfection with miR-448 mimic or inhibitor for 48 h, and then MMP-13 expression was determined at the mRNA and protein levels using PCR or ELISA. IL-1β stimulation promoted chondrocytes to secrete MMP-13 protein in the culture supernatant, and downregulation of miR-448 significantly reduced the secretion of MMP-13 induced by IL-1β (Fig. 3a). Knockdown of miR-448 obviously reduced the increase in IL-1β-induced MMP-13 mRNA expression (Fig. 3b). Overexpression of miR-448 promoted IL-1β-stimulated expression of MMP-13 (Fig. 3c and d).

### MiR-448 directly targets matrilin-3

**Fig. 4:**
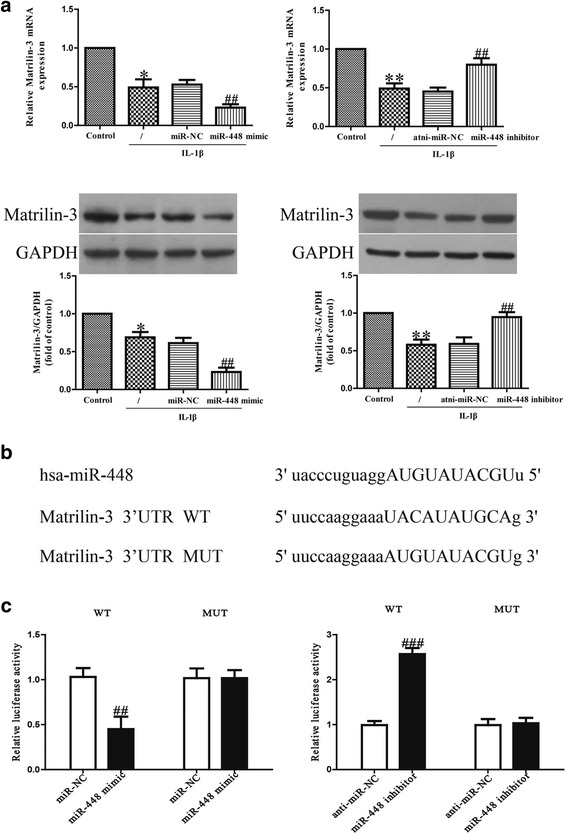
Matrilin-3 was a direct target of miR-448. Chondrocytes were treated with 5 ng/ml IL-1β for 24 h after transfection with miR-448 mimic or inhibitor. **a** The mRNA and protein levels of matrilin-3 were respectively determined using quantitative real-time PCR and western blot. Matrilin-3 expression was normalized to GAPDH. **b** Schematic representation of matrilin-3 3’UTRs showing putative miRNA target site. **c** The analysis of the relative luciferase activities of matrilin-3-WT and matrilin-3-MUT. All data are presented as means ± SEM, *n* = 4. *p < 0.05, **p < 0.01 vs. control; ^##^*p* < 0.01, ^###^*p* < 0.001 vs. vehicle + IL-1β or miR-NC or anti-miR-NC

The online database microRNA.org was used to predict the target gene. It showed that matrilin-3 is a direct target of miR-448. The expressions of matrilin-3 at the mRNA and protein levels were assessed using PCR and western blot assays in chondrocytes transfected with the miR-448 inhibitor or mimic. The results indicate that after IL-1β exposure, the mRNA level of matrilin-3 was significantly lower than with overexpression of miR-448 (Fig. 4a), but significantly higher in the cells with inhibition of miR-448 compared with the IL-1β-treated cells.

To further confirm whether matrilin-3 was a direct target of miR-448, we constructed a luciferase reporter vector containing the WT 3’-UTR matrilin-3, and the putative miR-448 binding site in the matrilin-3 3’-UTR was mutated (MUT 3’-UTR matrilin-3; Fig. 4b). The findings indicated that overexpression or knockdown of miR-448 respectively suppressed or enhanced the luciferase activity in chondrocytes transfected with the WT 3’-UTR of matrilin-3 (Fig. 4c). The MUT 3’-UTR of matrilin-3 blocked the effect of miR-448. These data demonstrate that matrilin-3 is directly and negatively regulated by miR-448, and that miR-448 might function in osteoarthritis through regulation of matrilin-3.

### Inhibition of matrilin-3 is essential for protective effect of miR-448 on IL-1β-induced chondrocyte injury

**Fig. 5:**
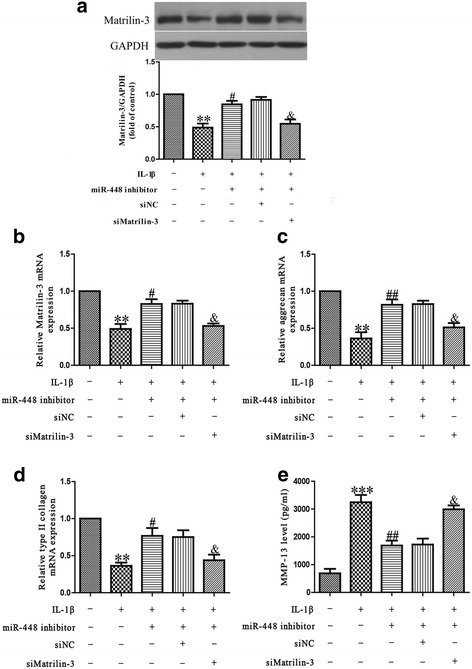
Matrilin-3 was involved in the effects of miR-448 on IL-1β-induced cartilage degradation in chondrocytes. Chondrocytes were transfected with either miR-448 inhibitor or si-matrilin-3, and then treated with 5 ng/ml IL-1β for 24 h. **a**, **b** The mRNA and protein levels of matrilin-3 were respectively determined using quantitative real-time PCR and western blot. The mRNA levels of aggrecan (**c**) and type II collagen (**d**) were determined using quantitative real-time PCR. GAPDH was detected as a loading control. **e** The expression level of secreted MMP-13 protein was determined by ELISA. All data are presented as means ± SEM, *n* = 4. **p < 0.01, ***p < 0.001 vs. control; ^#^*p* < 0.05, ^##^*p* < 0.01 vs. vehicle + IL-1β; ^&^*p* < 0.05 vs. IL-1β + miR-448 inhibitor

To explore whether downregulation of miR-448 protected IL-1β-induced chondrocytes in a manner dependent on the matrilin-3 level, chondrocytes were co-transfected with miR-448 inhibitor and si-matrilin-3, and then stimulated with IL-1β for 24 h. Our findings showed that the IL-1β-inhibited expression of matrilin-3 in chondrocytes was significantly upregulated after transfection with the miR-448 inhibitor, and knockdown of matrilin-3 could block the effect of the miR-448 inhibitor on matrilin-3 expression (Fig. 5a and b). Silencing miR-448 attenuated the IL-1β-induced decrease in aggrecan and type II collagen mRNA expressions and the increase in MMP-13 protein expression, but this effect was was blocked by the addition of si-matrilin-3 (Fig. 5c–e). This clearly confirms that inhibition of miR-448 improved IL-1β-induced decreases in collagen synthesis of chondrocytes by increasing matrilin-3 expression.

### The effects of miR-448 on osteoarthritis chondrocytes

**Fig. 6:**
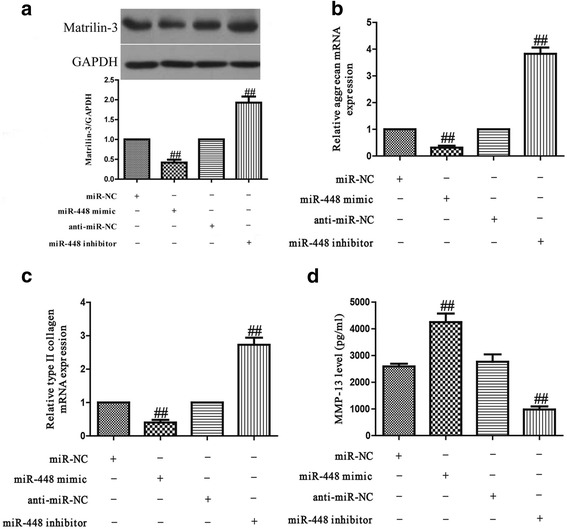
Effects of miR-448 on osteoarthritis chondrocytes. Osteoarthritis chondrocytes were transfected with miR-448 mimic or inhibitor. **a** The protein level of matrilin-3 were determined using western blot. The mRNA levels of aggrecan (**b**) and type II collagen (**c**) were determined using quantitative real-time PCR. GAPDH was detected as a loading control. **d** The expression level of secreted MMP-13 protein was determined using ELISA. All data are presented as means ± SEM, *n* = 4. ^##^*p* < 0.01 vs. miR-NC or anti-miR-NC

We transfected human osteoarthritis chondrocytes with an miR-448 mimic or inhibitor, expressions of matrilin-3, aggrecan, type II collagen and MMP-13 were determined using western blot, quantitative real-time PCR and ELISA assays as appropriate. The findings suggested that introduction of miR-448 significantly decreased the protein expression of matrilin-3 and the mRNA levels of aggrecan and type II collagen, and enhanced the secreted protein expression of MMP-13 compared with osteoarthritis chondrocytes transfected with miR-NC (Fig. 6). Knockdown of miR-448 significantly increased the expressions of matrilin-3, aggrecan and type II collagen, and decreased the expression of MMP-13 compared with osteoarthritis chondrocytes transfected with anti-miR-NC (Fig. 6).

## Discussion

Osteoarthritis is a complex inflammatory disease involving the whole joint [16]. MiRNAs have been demonstrated to have functional roles in its pathogenesis [17]. Since IL-1β plays an important role in the pathogenesis of osteoarthritis, it has often been used to establish an in vitro osteoarthritis model in chondrocytes [18, 19]. In studies examining responses to IL-1β, the processes of disrupted cartilage homeostasis have been found to be regulated by several miRNAs, including miR-145, miR-139, miR-9, miR-193b and miR-320 [20–24]. To further our understanding of the disease and find potential treatments, it is critical to characterize the gene networks regulated by these miRNAs.

It has been reported that miR-448 acts as a tumor suppressor in many cancers. For example, miR-448 inhibits invasion and EMT by downregulating ROCK2 in hepatocellular carcinoma [25]. Yu et al. demonstrated that miR-448 can suppress the metastasis of pancreatic ductal adenocarcinoma by targeting the JAK1/STAT3 pathway [26]. Overexpression of miR-448 suppresses proliferation and invasion by regulating IGF1R in colorectal cancer cells. It is thus interesting to examine whether this miRNA also plays an important role in other pathologies, such as osteoarthritis.

Our data show that the level of miR-448 was significantly higher in osteoarthritis cartilage than in normal cartilage, and significantly increased in response to IL-1β stimulation. These results suggest that miR-448 potentially contributes to the pathogenesis of osteoarthritis.

During the development of osteoarthritis, IL-1β plays a key role in cartilage degradation [27]. Articular cartilage ECM regulates multiple biological processes that are important for the repair and homeostasis of cartilage [28]. The impairment of joint function is induced by the continuous loss of cartilage ECM, which is involved in the progression of osteoarthritis [29]. Several studies have confirmed that IL-1β greatly influences the production of the ECM components in chondrocytes, leading to interference with the synthesis of key structural proteins such as aggrecan and type II collagen. Moreover, IL-1β affects MMP synthesis in the chondrocytes, including that of MMP-13 [30].

Consistent with earlier results, our data also showed that IL-1β induced a decrease in the expressions of aggrecan and type II collagen, and an increase in MMP-13 expression. A functional analysis of miR-448 was done to confirm whether it regulated the expressions of ECM components. Modulation of miR-448 efficiently affected IL-1β-stimulated degradation and synthesis of ECM in osteoarthritis chondrocytes. Knockdown of miR-448 could reverse IL-1β-induced impairment of aggrecan and type II collagen, and evidently inhibited the increase in IL-1β-induced MMP-13 expression. Conversely, introduction of miR-448 aggravated IL-1β-induced decreased expression of aggrecan and type II collagen, and further enhanced IL-1β-induced MMP-13 production. These results indicated that the downregulation of miR-448 inhibited the progression of osteoarthritis by promoting chondrocyte ECM synthesis.

Matrilin-3 is a non-collagenous oligomeric ECM protein, belonging to one of the four members of the matrilin family [31–34]. As an ECM protein, matrilin-3 can interact with collagen fibrils, multiple proteoglycans and other glycoproteins, playing a critical structural role in forming a filamentous matrix network [35]. Mutations of matrilin-3 in humans are closely related to many kinds of skeletal disease, including osteoarthritis [36–39], emphasizing its importance in the development and homeostasis of cartilage. Furthermore, it has been identified that miR-448 directly regulated matrilin-3, which was an essential factor for chondrocyte homeostasis, by binding the predicted seed sites, contributing to the inhibition of matrilin-3 expression at the mRNA level.

It was previously demonstrated that matrilin-3 modulates the balance between ECM synthesis and degradation of articular chondrocyte via upregulation of the expressions of aggrecan and type II collagen, and downregulation of MMP-13 expression [15]. In this study, the data show that matrilin-3 was evidently reduced in osteoarthritis cartilage compared to normal cartilage. Consequently, it was speculated that increased miR-448 regulated the progression of osteoarthritis via decreasing matrilin-3 expression. The data indicate that upregulation of miR-448 markedly reduced the mRNA expression of matrilin-3. However, the downregulation of miR-448 upregulated the matrilin-3 mRNA level. The upregulation of aggrecan and type II collagen and downregulation of MMP-13, which were stimulated by knockdown of miR-448, were significantly reduced by decreasing the matrilin-3 expression.

The results suggest that introduction of miR-448 downregulated matrilin-3, thereby inhibiting ECM synthesis of chondrocytes and resulting in the progression of osteoarthritis. Eventually, to confirm these results, the effects of miR-448 were studied in osteoarthritis chondrocytes. As expected, miR-448 also had similar effects on IL-1β-induced chondrocytes. Based on our findings, we believe that the levels of miR-448 and matrilin-3 could be considered as biomarkers for a diagnostic index of osteoarthritis.

## Conclusions

Our findings demonstrate that miR-448 inhibition directly upregulated matrilin-3 expression, protecting IL-1β-induced and osteoarthritis chondrocytes from ECM degradation. MiR-448 acts as a negative regulator of the IL-1β-induced disturbance to the metabolism of human chondrocytes, and this occurs partially through the inhibition of matrilin-3.

## Abbreviations

ECM: Extracellular matrix
miRNA: microRNA
MMP-13: Matrix metalloproteinase 13
